# Cell-Penetrating Peptide-Mediated siRNA Targeting of LDHC Suppresses Tumor Growth in a Triple-Negative Breast Cancer Zebrafish Xenograft Model

**DOI:** 10.3390/pharmaceutics18010078

**Published:** 2026-01-07

**Authors:** Hanan Qasem, Adviti Naik, Tricia Gomez, Janarthanan Ponraj, Umar Jafar, Martin Sikhondze, Remy Thomas, Khaled A. Mahmoud, Julie Decock

**Affiliations:** 1 College of Health and Life Sciences (CHLS), Hamad Bin Khalifa University (HBKU), Qatar Foundation (QF), Doha P.O. Box 34110, Qatar; hqasem@hbku.edu.qa (H.Q.); umja44783@hbku.edu.qa (U.J.); 2 Translational Oncology Research Center, Qatar Biomedical Research Institute (QBRI), Hamad Bin Khalifa University (HBKU), Qatar Foundation (QF), Doha P.O. Box 34110, Qatar; advitin@andrew.cmu.edu (A.N.); msikhond@alumni.cmu.edu (M.S.);; 3 Qatar Environment and Energy Research Institute (QEERI), Hamad Bin Khalifa University (HBKU), Qatar Foundation (QF), Doha P.O. Box 34110, Qatar; tgomez@hbku.edu.qa (T.G.); jponraj@hbku.edu.qa (J.P.); kmahmoud@hbku.edu.qa (K.A.M.)

**Keywords:** LDHC, Lactate Dehydrogenase C, CPP, cell penetrating peptide, siRNA therapeutics, triple negative breast cancer

## Abstract

**Background**: Lactate Dehydrogenase C (LDHC) is a promising therapeutic target due to its highly tumor-specific expression, immunogenicity, and oncogenic functions. We previously showed that *LDHC* silencing in triple-negative breast cancer (TNBC) cells enhances treatment response to DNA-damage response-related drugs, supporting its therapeutic potential. However, no selective LDHC inhibitors exist, highlighting the need for innovative targeting strategies. **Methods**: We assessed the physicochemical properties and evaluated the delivery efficiency, anti-tumor activity, and safety of four cell-penetrating peptides (CPPs)—R10, 10R-RGD, cRGD-10R, and iRGD-10R—for siRNA-mediated *LDHC* silencing in TNBC. Clonogenic assays were used to evaluate effects on olaparib sensitivity, and TNBC zebrafish xenografts were utilized to study in vivo anti-tumor activity. **Results**: All CPP:siRNA complexes formed uniform nanocomplexes (129–168 nm) with low polydispersity indices (<0.25) and positive zeta potentials (+6.47 to +29.6 mV). Complexes remained stable in human serum for 24 h and showed no significant cytotoxicity in TNBC and non-cancerous cell lines. The 10R-RGD and cRGD-10R:siLDHC complexes achieved 40% LDHC protein knockdown, reduced TNBC clonogenicity by 30–36%, and enhanced olaparib sensitivity. Treatment of TNBC zebrafish xenografts with 10R-RGD or cRGD-10R:siLDHC complexes significantly reduced tumor growth by approximately 50% without major toxicity. **Conclusions**: These results demonstrate that CPP-mediated siRNA delivery enables selective *LDHC* silencing with tumor growth inhibition in triple-negative breast cancer models. This approach represents a novel, effective, and safe proof-of-concept therapeutic strategy to target LDHC, with potential translational relevance as a standalone therapy or in combination with common anti-cancer drugs.

## 1. Introduction

Breast cancer is the most prevalent cancer and the leading cause of mortality among women worldwide [1]. Triple-negative breast cancer (TNBC), characterized by the lack of expression of the estrogen receptor, progesterone receptor, and Human Epidermal Growth Factor Receptor 2 (Her2), represents 10–20% of breast cancer cases, primarily affects young women, and is associated with a poor prognosis and high metastatic potential [2,3]. Currently, the treatment options for patients with TNBC include chemotherapy, radiotherapy, and, more recently, immunotherapy. While TNBC patients exhibit early pathological responses to chemotherapy, unfortunately, a significant portion of patients develop resistance and disease recurrence.

In recent years, cancer treatment has shifted away from a one-size-fits-all approach toward precision medicine, in which traditional treatments such as surgery, chemotherapy, and radiotherapy are complemented by personalized drug therapies [2,4]. Recent studies suggest that combining molecular targeting with chemotherapy represents a promising approach to treating TNBC [5]. For instance, the addition of mTOR inhibitors, such as temsirolimus or everolimus to doxorubicin and bevacizumab has significantly improved the objective response rate in TNBC patients with tumors that display aberrant activation of the PI3K/mTOR pathway [6]. In addition, combination treatment with carboplatin and the PARP inhibitor olaparib is associated with an overall response rate of 88% in TNBC patients with *BRCA* mutations. In this context, specific targeting of cancer/testis antigens (CTAs) has gained interest thanks to their highly tumor-specific expression, immunogenic properties, and multifaceted roles in promoting cancer hallmarks [7,8,9,10]. Aberrant expression of Lactate Dehydrogenase C (LDHC) is observed in different types of cancer, where it is associated with tumor progression, metastasis, and poor prognosis [11,12,13]. Previously, we demonstrated that knockdown of *LDHC* reduces the long-term survival of breast tumor cells, particularly TNBC cells, by dysregulating cell cycle progression and impairing the DNA damage response pathway [14]. More specifically, we showed that knockdown of *LDHC* induced genomic instability and mitotic catastrophe, reduced the expression of DNA damage sensor molecules, increased the expression of the DNA damage marker phospho-γH2AX, and disrupted microtubule organization. As a result of enhanced mitotic catastrophe, LDHC-deficient cancer cells exhibited a higher rate of cell death and mitotic slippage through the dysregulation of multiple cell cycle checkpoints. LDHC can thus be included in the expanding group of CTAs involved in regulating genomic integrity. Moreover, we found that targeting LDHC greatly improved treatment response to commonly used DNA damage response-related drugs such as cisplatin and olaparib.

Gene therapy using RNA interference (RNAi)-based drugs has shown remarkable progress with numerous tumor-related RNAi drugs undergoing clinical trials [15]. For instance, RNAi therapeutics targeting anti-apoptosis genes, oncogenes, and tumor signaling molecules such as Bcl-2, MYC, KRAS, AKT1, and STAT3 have entered phase II trials. Despite promising preclinical outcomes, RNAi-based therapy has yet to transition into clinical practice as several challenges remain to be addressed, including stability, targeting ability, off-target effects, and toxicity. Advances in the development of cell-penetrating peptides as delivery systems for RNAi-based therapy have aided in addressing some of these challenges. The use of CPPs improves RNAi serum stability and internalization efficiency. Additionally, CPPs containing the arginine–glycine–aspartic acid (RGD) tripeptide enhance tumor specificity through binding of integrins αvβ3 (INTαVβ3) [16]. The use of CPPs has demonstrated efficient delivery of siRNA cargo with notable anti-tumor activity. For example, treatment with cyclic-RGD:siEGFR significantly decreased tumor size in glioblastoma (U87MG) xenograft mice [17]. Furthermore, multiple studies have reported that CPPs incorporating an internalizing RGD peptide (iRGD) enhance tumor penetration and improve anti-cancer therapeutic effects [18,19]. In this study, we investigated the delivery efficacy, anti-tumor activity, and safety of four distinct CPPs targeting LDHC, including polyarginine (R10), 10R-linear RGD (10R-RGD), cyclic-RGD-10R (cRGD-10R), and internalizing RGD-10R (iRGD-10R). All CPPs formed uniform, positively charged siRNA nanocomplexes with good serum stability, efficient cellular uptake, and effective LDHC knockdown in triple-negative breast cancer cell lines. Each of the CPP:siRNA nanocomplexes was associated with a favorable safety profile in non-cancerous and cancerous cell lines. Administration of the 10R-RGD and cRGD-10R peptide–siLDHC complexes decreased long-term tumor cell survival and improved chemotherapy responses in vitro. Moreover, 10R-RGD and cRGD-10R-based delivery of siLDHC significantly reduced tumor growth in TNBC zebrafish xenografts in the absence of toxicity.

## 2. Materials and Methods

### 2.1. Cell Penetrating Peptides (CPPs) and siRNA

CPPs were synthesized by ThermoFisher Scientific (Waltham, MA, USA) using solid-phase peptide synthesis, and purity was determined by high-performance liquid chromatography. CPP sequences, purity, and molecular weight are listed in Table 1. CPPs were resuspended in RNase/DNase-free water at 1 mg/mL. Accell siLDHC#1 (A-008759-14-0020), siLDHC#2 (A-008759-15-0020), and siCTRL1 (D-001910-20) were obtained from Dharmacon (Lafayette, CO, USA) and resuspended at 100 µM.

### 2.2. CPP:siRNA Complex Preparation

CPP (1 mg/mL stock) and siRNA (100 µM stock) were mixed at various peptide–siRNA molar ratios (2.5:1, 5:1, 10:1) in 50 µL of Ultra-Pure DNase/RNase-free water. After 45 min incubation at room temperature, Opti-MEM I Reduced Serum medium (ThermoFisher Scientific, Waltham, MA, USA, #11058-021) was added to achieve a final siRNA concentration of 200 nM and CPP concentration of 30–60 nM.

### 2.3. Gel Retardation Assay

CPP–siRNA complex solutions at various peptide–siRNA molar ratios were supplemented with 1.25× formamide loading dye, and resolved on a 20% native polyacrylamide gel electrophoresis for 120 min at 120 V using 1× Tris-Boric-EDTA buffer, alongside a 10 kb DNA ladder (ThermoFisher Scientific, Waltham, MA, USA, #SM033). The gels were stained with SYBR gold (ThermoFisher Scientific, Waltham, MA, USA, #S11494) for 20 min and analyzed under UV light using the ChemiDoc XRS+ Imaging system (Bio-Rad, Hercules, CA, USA, RRID:SCR_019690). Unconjugated or naked siRNA was used as a control.

### 2.4. Physicochemical Characterization of CPP:siRNA Nanocomplexes

Particle size, zeta potential, and polydispersity index were measured for each CPP:siRNA complex, diluted in 1 mL Ultra-Pure DNAse/RNAse-free water, using a Zetasizer Ultra ZSU5700 (Malvern Instruments Inc., Worcestershire, UK) at a wavelength of 677 nm with a constant angle of 90° at 37 °C. Additionally, complex morphology and size were analyzed by transmission electron microscopy using the FEI Talos F200X G2 microscope (ThermoFisher Scientific, Waltham, MA, USA, RRID:SCR_019907). Briefly, 10 µL of sample suspension was applied onto a 300-mesh carbon-coated copper grid, negatively stained with uranyl acetate (Electron microscopy science, Hatfield, CA, USA, #22405), and air-dried prior to collecting images using the FEI Talos F200C Transmission Electron Microscope (ThermoFisher Scientific, Waltham, MA, USA, RRID:SCR_019903).

### 2.5. Serum Stability Assay

CPPs were complexed with siLDHC#2 at a 5:1 peptide–siRNA molar ratio for 45 min at room temperature. Next, CPP:siRNA complexes and naked siRNA were incubated at 37 °C with 50% normal human serum (ThermoFisher Scientific, Waltham, MA, USA, #31876) or Ultra-Pure DNAse/RNAse-free water for 6, 12, 24, 48, or 72 h and frozen at −20 °C. Samples were centrifuged at 10,000 rpm for 5 min at 4 °C, and pellets were resuspended in 15 μL of Ultra-Pure DNAse/RNAse-free water. Finally, samples were incubated with proteinase K (0.006 mM), CaCl_2_ (0.3 mM), and Tris-HCl (3 mM, pH  =  7.0) for 5.5–6 h at 37 °C. Formamide loading buffer was added to the samples alongside a 10 kb DNA ladder (ThermoFisher Scientific, Waltham, MA, USA, #SM0333) after which electrophoresis was performed using native 20% PAGE for 120 min at a constant voltage of 120 V. Gels were stained with SYBR Gold (ThermoFisher Scientific, Waltham, MA, USA, S11494) for 20 min, and analyzed using ChemiDoc XRS+ Imaging system (Bio-Rad, Hercules, CA, USA, RRID:SCR_019690).

### 2.6. Cell Culture

MDA-MB-453 (RRID:CVCL_0418), MDA-MB-468 (RRID:CVCL_0419), BT-549 (RRID:CVCL_1092), DU4475 (RRID:CVCL_1183), HUVEC (RRID:CVCL_9Q53) were purchased from the American Tissue Culture Collection (ATCC, Manassas, VA, USA), and IMR-90 (RRID:CVCL_0347) from European Collection of Authenticated Cell Cultures (ECACC, Porton Down, UK). MDA-MB-468 and MDA-MB-453 cells were maintained in Dulbecco’s Minimum Essential Media (DMEM, ThermoFisher Scientific, Waltham, MA, USA, #10569-010) supplemented with 10% *v*/*v* fetal bovine serum (FBS, ThermoFisher Scientific, Waltham, MA, USA, #10082-147), 50 U/mL Penicillin, and 50 µg/mL Streptomycin (ThermoFisher Scientific, Waltham, MA, USA, #15140-122). BT-549 cells were cultured in ATCC-formulated Roswell Park Memorial Institute (RPMI)-1640 medium (ThermoFisher Scientific, Waltham, MA, USA, #A10491-01) supplemented with 10% (*v*/*v*) FBS (ThermoFisher Scientific, Waltham, MA, USA, #10082-147), 50 U/mL penicillin and 50 µg/mL streptomycin (ThermoFisher Scientific, Waltham, MA, USA, #15140-122), and 0.023 IU/mL insulin (Sigma-Aldrich, St. Louis, MO, USA, #11070-73-8). DU4475 cells were maintained in ATCC-formulated Roswell Park Memorial Institute (RPMI)-1640 medium (ThermoFisher Scientific, Waltham, MA, USA, A10491-01) supplemented with 10% (*v*/*v*) FBS (ThermoFisher Scientific, Waltham, MA, USA, #10082-147), 50 U/mL Penicillin, and 50 µg/mL Streptomycin (ThermoFisher Scientific, Waltham, MA, USA, #15140-12). HUVEC cells were maintained in EBM-2 (Lonza, Basel, Switzerland, #cc-3156) supplemented with EGM^TM^-2 singleQuots supplements (Lonza, Basel, Switzerland, # CC-4176) and using a collagen-coated flask (ThermoFisher Scientific, Waltham, MA, USA, #132606). IMR-90 cells were cultured in Minimum Essential Media (MEM, ThermoFisher Scientific, Waltham, MA, USA, #41090-028) supplemented with 10% *v*/*v* FBS (ThermoFisher Scientific, Waltham, MA, USA, #10082-14), 50 U/mL Penicillin and 50 µg/mL Streptomycin (ThermoFisher Scientific, Waltham, MA, USA, #15140-122), 1% sodium pyruvate (ThermoFisher Scientific, Waltham, MA, USA, #11360-039), 1% non-essential amino acids (ThermoFisher Scientific, Waltham, MA, USA, #11140-050). All cell lines were maintained at 37 °C and 5% CO_2_ in a humidified incubator. Regular mycoplasma testing was conducted using a polymerase chain reaction (PCR)-based detection assay. Early passage cells (<P10) were used for all experiments.

### 2.7. Cellular Uptake of CPP:siRNA Nanocomplexes

To enable visualization of cellular uptake of the CPP:siRNA complexes in cancer cells, siRNA was pre-labeled with Cy™3 using the Silencer™ siRNA Labeling Kit (ThermoFisher Scientific, Waltham, MA, USA, #2960050). A total of 1 × 10^5^ MDA-MB-468 breast cancer cells were plated per well in a 12-well plate and left overnight at 37 °C and 5% CO_2_. Next, cells were washed once with DPBS (ThermoFisher Scientific, Waltham, MA, USA, #14190-094) and pre-incubated in Opti-MEM I Reduced Serum medium (ThermoFisher Scientific, Waltham, MA, USA, #11058-021) for 30 min, after which 500 µL of CPP:siRNA (400 nM siRNA in Opti-MEM I Reduced Serum medium) was added to each well. Reduced serum conditions were used to maximize cellular delivery and gene silencing under controlled conditions where serum proteins do not interfere with complex–cell interactions. After 6 h, 500 µL of complete, antibiotic-free DMEM media was added (final siRNA concentration = 200 nM). In parallel, cells were incubated with either 100 pmol of siRNA alone (naked siRNA control) or were transfected with siRNA using RNAiMAX (ThermoFisher Scientific, Waltham, MA, USA, #13778075) according to the manufacturer’s guidelines. After 72 h, cellular uptake of CPP:siRNA-Cy3 complexes was visualized using the Olympus IX73 inverted microscope (Olympus, Tokyo, Japan, RRID:SCR_020346) at 10× magnification.

### 2.8. Flow Cytometry Analysis of Integrin αvβ3 Expression

A range of breast cancer and non-cancerous cell lines with varying expression of integrin αvβ3 was selected to assess RGD-mediated cellular uptake and toxicity of CPP:siRNA complexes. Integrin αVβ3 expression was analyzed using flow cytometry, real-time qRT-PCR, and Western blotting. For flow cytometry, a total of 1 × 10^5^ cells were incubated with 100 µL stain buffer (BD Biosciences, Franklin Lakes, NJ, USA, #554656), supplemented with 5 μL of human FcR Blocking reagent (Miltenyi Biotec, Bergisch Gladbach, Germany, #130-059-901). After 15 min incubation at 4 °C, cells were incubated with mouse anti-human integrin αVβ3 BV421 conjugated antibody (BD Biosciences, Franklin Lakes, NJ, USA, #744088, RRID:AB_2741983) at 1:20 for one hour, followed by two washes with DPBS (ThermoFisher Scientific, Waltham, MA, USA, #14190-094) at 300× *g* for 5 min at room temperature. Finally, integrin αVβ3 expression was analyzed on the BD LSRFortessa X-20 instrument (BD Biosciences, Franklin Lakes, NJ, USA, RRID:SCR_025285). For each sample, 10,000 events were recorded, and further analysis was performed using FlowJo™ Software (BD Biosciences, Franklin Lakes, NJ, USA, version 10.8).

### 2.9. Expression Analysis of LDHC and Integrins Using Quantitative Real-Time Reverse Transcription Polymerase Chain Reaction (qRT-PCR)

To assess the effect of CPP:siRNA treatment on *LDHC* expression, total RNA was isolated 72 h after treatment using the RNeasy Mini kit (Qiagen, Hilden, Germany, #74106). In addition, total RNA was extracted from a range of breast cancer and non-cancerous cell lines to determine the expression of *integrin αV* and *integrin β3*. RNA quantity and purity were assessed using A260/A280 and A260/A230 measurements on a Nanodrop2000 spectrophotometer (ThermoFisher Scientific, Waltham, MA, USA, RRID:SCR_018042). Next, cDNA was synthesized from 1 µg of total RNA using the M-MLV Reverse Transcriptase kit (ThermoFisher Scientific, Waltham, MA, USA, #28025-013) according to the manufacturer’s guidelines. *LDHC* expression was quantified using a specific 5′FAM-3′MGB TaqMan gene expression primer/probe set (Hs00255650_m1, Applied Biosystems, Foster City, CA, USA). The mRNA expression of integrin αV and integrin β3 was quantified using 100 ng of cDNA, specific SYBR-based qPCR primers (integrin αv F: 5-GGGACTCCTGCTACCTCTGT-3, integrin αV R: 5-GAAGAAACATCCGGGAAGACG-3, integrin β3 F: 5-ACTGGCAAGGATGCAGTGAA-3, and integrin β3 R: TTGGACACTCTGGCTCTTC-3) and the PowerUp SYBR Green master mix (Applied Biosystems, Foster City, CA, USA, #A25742). All reactions were performed on the QuantStudio 7 Real-time PCR instrument (Applied Biosystems, Foster City, CA, USA, RRID:SCR_020245). Expression levels were normalized to the housekeeping gene *RPLPO* (TaqMan primer/probe 4333761F or SYBR primers F: TCCTCGTGGAAGTGACATCG, R: TGGATGATCTTAAGGAAGTAGTTGG) and differential gene expression was calculated using the 2^−ΔΔCt^ method.

### 2.10. Western Blotting of LDHC and Integrins

Western blotting was used to determine protein expression of LDHC, integrin αV, and integrin β3 in various cell lines. Approximately 5 × 10^5^ MDA-MB-468 cells were plated in 6-well plates, followed by incubation with CPP:siRNA (final siRNA conc = 200 nM) for 72 h after which protein lysates were isolated using RIPA buffer (ThermoFisher Scientific, Waltham, MA, USA, #89900) supplemented with a HALT protease and phosphatase inhibitor cocktail (ThermoFisher Scientific, Waltham, MA, USA, #87786). Protein lysates were centrifuged for 30 min at 20,000× *g*, and protein content of supernatants was determined using the BCA protein assay (ThermoFisher Scientific, Waltham, MA, USA, #23225). Protein samples were reduced and denatured in 4× Laemmli sample buffer (Bio-Rad, Hercules, CA, USA, #161-07470), loaded onto a 4–15% TGX gel (Bio-Rad, Hercules, CA, USA, #4561084), and transferred onto polyvinylidene fluoride (PVDF) membrane (Bio-Rad, Hercules, CA, USA, #1704156). Membranes were blocked in 5% non-fat dried milk/Tris-buffered saline with 0.1% Tween-20, washed and incubated overnight at 4 °C with the following primary antibodies diluted in blocking buffer; rabbit anti-human LDHC (Abcam, Cambridge, UK, #ab52747, RRID: AB_880686, 1:1000), rabbit anti-human integrin αV (Cell signaling Technology, Danvers, MA, USA, #4711, RRID:AB_2128178, 1:1000), rabbit anti-human integrin β3 (Cell signaling Technology, Danvers, MA, USA, #13166, RRID:AB _27981361, 1:1000) and rabbit anti-human β-actin (Cell signaling Technology, Danvers, MA, USA, #4970, RRID:AB_2223172, 1:1000). Next, membranes were washed, incubated with horseradish Peroxidase (HRP)-linked rabbit secondary antibody (Cell signaling Technology, Danvers, MA, USA, #7074, RRID:AB_2099233, 1:5000) for 1 h at room temperature, and proteins were detected by ECL Plus (ThermoFisher Scientific, Waltham, MA, USA, #32209) using the ChemiDoc XRS+ Imaging system (Bio-Rad, Hercules, CA, USA, RRID:SCR_019690). Image acquisition and densitometry analysis were performed using the IMAGE LAB software v 6.1 (Bio-Rad, Hercules, CA, USA, RRID:SCR_014210).

### 2.11. Cytotoxicity Assay

To assess toxicity induced by CPP:siRNA treatment, breast cancer and non-cancerous cells were seeded at 1 × 10^4^ cells per well in an opaque 96-well plate and allowed to adhere overnight at 37 °C and 5% CO_2_. Next, 100 µL CPP:siRNA complexes (200 nM siRNA, 50% *v*/*v* Opti-MEM I Reduced Serum medium and complete, antibiotic-free DMEM media) were added for 72 h at 37 °C and 5% CO_2_. Cytotoxicity was assessed using the CellTiter-Glo^®^ reagent (Promega, Madison, WI, USA, #G7572) following the manufacturer’s guidelines, and luminescence was recorded using the GloMax^®^-Multi Detection system (Promega, Madison, WI, USA, RRID:SCR_0155675).

### 2.12. Clonogenic Assay

To determine the effect of CPP:siRNA complexes on the long-term survival of breast cancer cells as single agents or in combination with Olaparib (Selleck Chemicals, Houston, TX, USA, #AZD2281), 1 × 10^4^ MDA-MB-468 breast cancer cells were seeded per well in a 12-well plate. On the 2nd and 7th day after seeding, cells were treated with CPP:siRNA complexes (50% *v*/*v* Opti-MEM I Reduced Serum medium and complete, antibiotic-free DMEM media), and on the 11th day after seeding, cells were treated with olaparib (30 µM). The concentration of olaparib and treatment duration were chosen as previously described [10]. Then, on the 14th day, cells were washed with DPBS (ThermoFisher Scientific, Waltham, MA, USA, #14190-094) and stained with 1% crystal violet (Sigma-Aldrich, St. Louis, MO, USA, #C6158) in 25% methanol. Excess stain was washed away, and crystal violet was eluted with 10% sodium dodecyl sulfate (SDS), and absorbance was measured at 590 nm on the NanoQuant infinite F200 Pro instrument (Tecan, Männedorf, Switzerland, RRID:SCR_024561).

### 2.13. Zebrafish Maintenance and Breeding

In vivo cytotoxicity and anti-tumor activity of CPP:siRNA complexes were determined using zebrafish. Wild-type AB zebrafish (Danio rerio) were maintained in standard conditions at the zebrafish laboratory at Sidra Medicine, Doha, Qatar. Adult zebrafish were set up for breeding, and embryos were collected and maintained in PTU-E3 media at 28.5 °C.

### 2.14. Zebrafish Embryo Toxicity Test

CPP:siRNA complexes (final siRNA concentration 150, 200, 250 nM) were injected into single-cell stage wild-type AB zebrafish embryos. At 4 days post-fertilization (dpf), the survival rate and morphology of the injected zebrafish embryos were compared to those of untreated embryos using the Zeiss Stemi 2000-C Stereo microscope (Zeiss, Oberkochen, Germany, RRID:SCR_020924). Zebrafish morphology was defined as G1: severely affected, G2: mildly affected, and G3: normal.

### 2.15. Breast Cancer Zebrafish Xenograft Model

The anti-tumor activity of the 10R-RGD:siRNA and cRGD-10R:siRNA complexes was assessed using a breast cancer zebrafish xenograft model. A total of 1 × 10^6^ MDA-MB-468 breast cancer cells were pre-labeled with Vybrant™ CM-DiI Cell-Labeling Solution (ThermoFisher Scientific, Waltham, MA, USA, #V-22888) for 20 min at 37 °C. Next, 1 × 10^3^ Dil-labeled MDA-MB-468 cells (in 5 nL complete DMEM media) were injected into the yolk sac of 48 h post-fertilization (hpf) anesthetized zebrafish embryos using a Pico-Liter Microinjector (Warner Instrument, Holliston, MA, USA, #PLI-100A). After microinjection, zebrafish embryos were maintained in PTU-E3 medium using 24-well plates at 34 °C. At 24 h post-injection (hpi), the embryos were imaged using a Zeiss AXIO Zoom.V16 microscope (Zeiss, Oberkochen, Germany) at 100× magnification and 560 nm, and embryos were selected based on size and location of tumor engraftment for further experiments. Next, CPP:siRNA complexes (final siRNA concentration 200 nM in 25 µL of Ultra-Pure DNAse/RNAse-free water with peptide–siRNA molar ratio of 5:1) were injected into the heart of the selected embryos at 32 hpi. Finally, embryos were imaged at 72 hpi using the Zeiss AXIO Zoom.V16 microscope (Zeiss, Oberkochen, Germany, RRID:SCR_027090) at 100× magnification and 560 nm. A total of 60 z-stack images were acquired and processed into maximum intensity projection images using the black ZEN software v3.10 (Zeiss, RRID:SCR_018163). The background corrected fluorescence intensity was determined using both ImageJ (v1.54g, RRID:SCR_003070) and ZEN (v 3.10, RRID:SCR_013672) software. The change in fluorescence intensity was calculated as 100× (fluorescence intensity at 72 hpi-fluorescence intensity at 24 hpi)/(fluorescence intensity at 24 hpi).

### 2.16. Statistical Analysis

Normality of data was assessed using the Shapiro–Wilk test, and the one-way analysis of variance (ANOVA) or two-tailed unpaired *t*-test was used to compare groups. *p*-value ≤ 0.05 was defined as statistically significant. Data are represented as mean ± standard error of mean (SEM). Statistical analyses and data representation were performed using GraphPad prism v10.0.0 (San Diego, CA, USA, RRID:SCR_002798).

## 3. Results

### 3.1. Assessment of CPP:siRNA Complex Formation, Serum Stability, and Physicochemical Characterization of the Nanocomplexes

**Figure 1:**
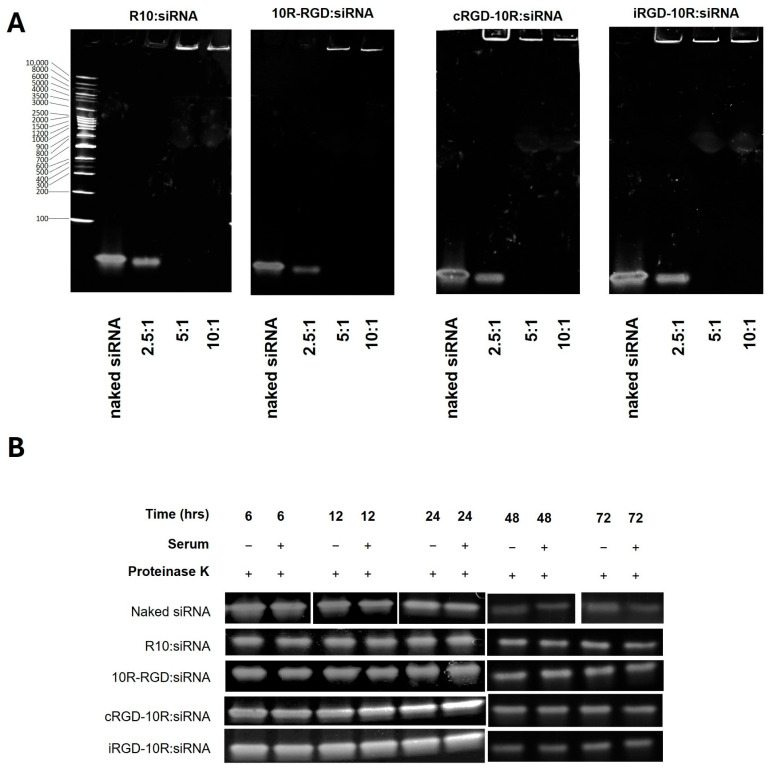
Assessment of CPP:siRNA complex formation and serum stability. (**A**) Visualization of R10:siLDHC#2, 10R-RGD:siLDHC#2, cRGD-10R:siLDHC#2, and iRGD-10R:siLDHC#2 complex formation at different peptide–siRNA molar ratios using gel retardation assay. (**B**) Serum stability assay of CPP:siLDHC#2 complexes (5:1 peptide–siRNA molar ratio) in 50% human serum.

Gel retardation assays were used to determine the optimal ratio required to encapsulate the siRNA with minimum release and maximum binding ability. LDHC siRNA was incubated with each of the four CPPs at different peptide–siRNA molar ratios (2.5:1, 5:1, 10:1). As shown in Figure 1A, robust complex formation was observed for all CPPs at peptide–siRNA molar ratios of 5:1 and 10:1. The ability of the CPP:siRNA complexes to protect the siRNA from degradation was assessed using a serum stability assay (using 5:1 peptide–siRNA molar ratio), which showed that siRNA integrity was maintained for up to 24 h in 50% human serum, followed by a gradual decline over time (Figure 1B).

**Figure 2:**
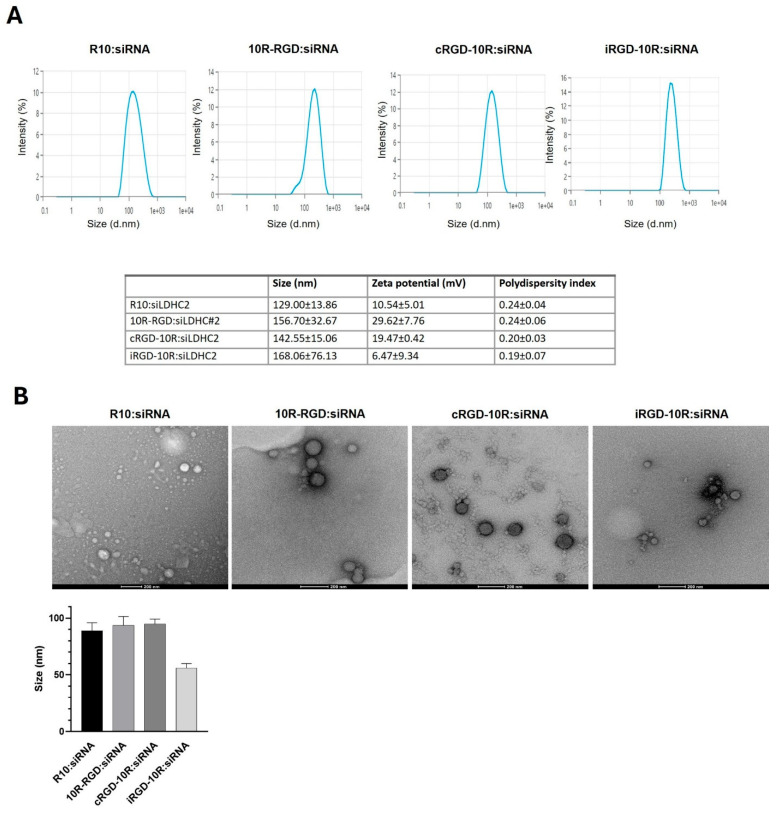
Physicochemical characterization of CPP:siRNA complexes: (**A**) Dynamic light scattering analysis of R10:siLDHC#2, 10R-RGD:siLDHC#2, cRGD-10R:siLDHC#2, and iRGD-10R:siLDHC#2 complexes at peptide–siRNA molar ratio of 5:1. Values represent mean and standard error of mean (±SEM) from three independent replicates. (**B**) Representative negative stain TEM images of CPP:siLDHC#2 complexes. Bar chart represents the means and standard error of mean (±SEM) from three independent experiments. Scale bar, 200 nm.

Dynamic light scattering analysis showed that the four distinct CPP:siRNA complexes exhibit an average hydrodynamic diameter between 129 and 168 nm, zeta potentials of 6.47 ± 9.34 to 29.62 ± 7.76 mV, and polydispersity indices below 0.25 (Figure 2A). Transmission electron microscopy revealed the formation of uniform, circular nanocomplexes of expected, smaller size (56–95 nm) compared to the hydrodynamic diameter measured by DLS (Figure 2B). Of note, we observed some aggregate formation of iRGD-10R complexes by transmission electron microscopy, which likely contributes to the larger hydrodynamic diameters measured by dynamic light scattering, reflecting a mixture of individual nanocomplexes and a few larger aggregates in solution.

### 3.2. CPP:siRNA Complexes Demonstrate Good Cellular Uptake in Breast Cancer Cells and Exhibit Favorable Safety Profile In Vitro

**Figure 3:**
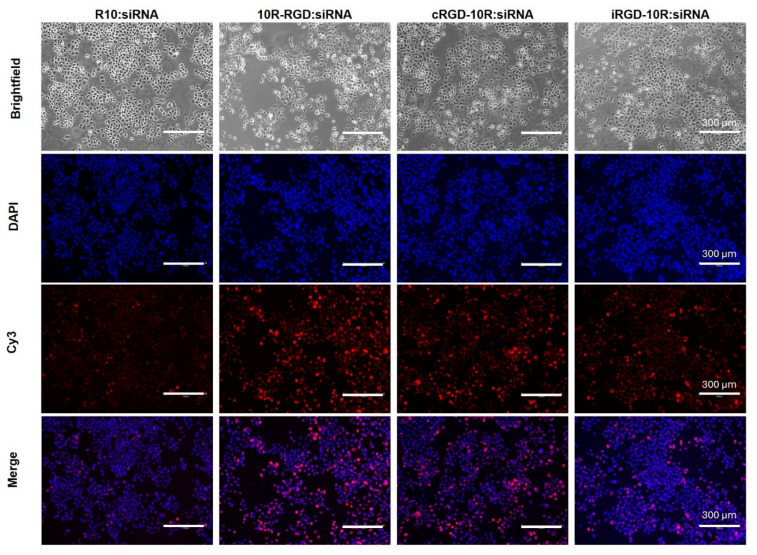
Cellular uptake of CPP:siRNA complexes in MDA-MB-468 breast cancer cells. Immunofluorescent imaging of Cy3-prelabeled CPP:siRNA complexes after 72 h incubation. Blue, DAPI; red, Cy3-labeled siRNA. Scale bar, 300 µm. Representative image of at least 3 biological replicates.

We assessed the cellular uptake efficacy of the various CPP:siRNA complexes using integrin αvβ3-positive MDA-MB-468 breast cancer cells (Figure 3). The 10R-RGD:siRNA and cRGD-10R:siRNA complexes demonstrated the highest cellular uptake efficiency compared to the R10:siRNA and iRGD-10R:siRNA complexes.

**Figure 4:**
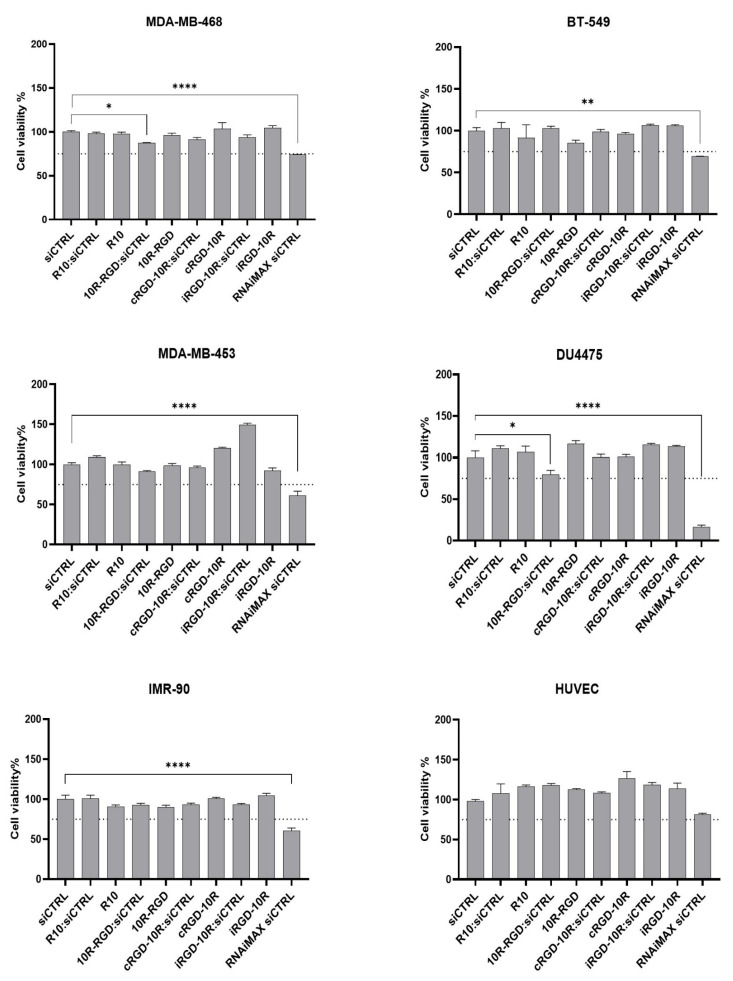
Treatment with CPP:siRNA complexes is associated with a favorable safety profile in vitro. Breast cancer and non-cancerous cell lines were treated with CPP:siRNA complexes (5:1 molar ratio) for 72 h, and cytotoxicity was determined using CellTiter-Glo luminescent cell viability assay. RNAiMAX-mediated transfection of naked siCTRL was used as a positive control for efficient cellular uptake, and CPPs alone were used to assess the toxicity of the peptides. The dotted line represents a decrease of 25% cell viability. Error bars represent the standard error of mean (±SEM) from three independent experiments. Statistical analysis to assess the reduction in cell viability was performed using the one-way ANOVA test with Dunnett correction. * *p* < 0.05, ** *p* < 0.01; **** *p* < 0.0001.

Next, a range of triple-negative breast cancer cell lines and two non-cancerous cell lines were used to investigate the potential presence of toxicity following treatment with the CPP:siRNA complexes. The cell lines were chosen based on their expression of integrins αv and β3 (Figure S1) to allow the simultaneous assessment of toxicity related to unspecific uptake or RGD-mediated cellular uptake. The breast cancer cell lines MDA-MB-468 and BT-549 were selected as integrin αvβ3-positive cancer cell line models, while the MDA-MB-453 and DU4475 breast cancer cell lines were chosen to represent integrin αvβ3-negative cancer cells. In addition, the integrin αvβ3-expressing IMR-90 and HUVEC cells were used to study the safety profiles of the CPP:siRNA complexes in non-cancerous cells. No significant cytotoxicity was observed in either the breast cancer cell lines or the non-cancerous cells, except for a minor toxicity of the 10R-RGD:siRNA complex in MDA-MB-468 cells (12% toxicity) and DU4475 cells (20% toxicity), suggesting that our CPP:siRNA complexes overall exhibit a favorable safety profile (Figure 4). Lipid-mediated cellular uptake of naked siRNA induced toxicity to varying degrees, as commonly seen using liposome transfection [20]. These findings are in line with previous studies reporting low cytotoxicity levels using CPPs [21,22].

### 3.3. siRNA Delivery by CPPs Efficiently Reduces LDHC Expression and Clonogenic Ability in Triple Negative Breast Cancer Cells In Vitro

**Figure 5:**
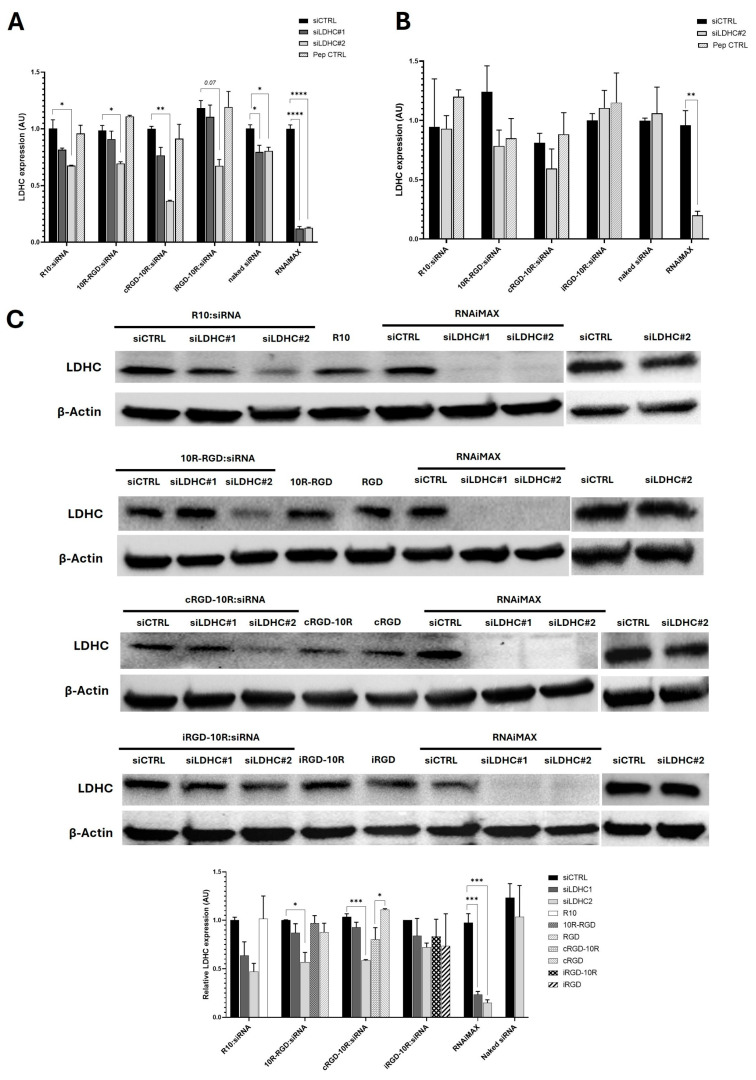
CPP:siRNA complexes demonstrate efficient LDHC knockdown in triple-negative breast cancer cells in vitro. (**A**) *LDHC* mRNA expression, normalized to *RPLPO*, of MDA-MB-468 and (**B**) BT-549 cells following treatment with CPP:siRNA complexes for 72 h. Pep CTRL—peptide control—refers to the respective peptides alone (R10, 10R-RGD, 10R-cRGD, and 10R-iRGD), which were used to assess non-specific changes in *LDHC* expression caused by the peptides alone. (**C**) Representative images of LDHC protein expression of MDA-MB-468 cells treated with CPP:siRNA complexes for 72 h. β-actin was used as a loading control. The bar chart represents densitometry values from three independent experiments (mean ± SEM). Statistical analysis was performed using the one-way ANOVA test with correction for comparison of more than two groups and a two-tailed unpaired *t*-test for comparison of two groups. * *p* < 0.05, ** *p* < 0.01, *** *p* < 0.001, **** *p* < 0.0001.

Given the robust complex formation, good cellular uptake, and low cytotoxicity of the CPP:siRNA complexes, we next sought to evaluate their LDHC knockdown efficiency in the integrin αvβ3-positive, LDHC-expressing triple-negative breast cancer cell lines MDA-MB-468 and BT-549. We found that the R10, 10R-RGD, and cRGD-10R peptides complexed with siLDHC#2 significantly reduced *LDHC* mRNA expression in MDA-MB-468 cells, with a borderline reduction in *LDHC* expression using the iRGD-10R peptide (Figure 5A). On the other hand, modest reductions in *LDHC* expression were seen in BT-549 cells (Figure 5B)**,** which is likely the result of their lower endogenous LDHC expression and likely reduced silencing efficiency [10]. Notably, we observed a greater reduction in *LDHC* expression with the CPP:siRNA complexes as compared to the naked siRNA. In particular, complexes containing siLDHC#2 exhibited higher knockdown efficiency, while showing comparable silencing as siLDHC#1 in lipid-based transfection. This is likely due to more favorable nanocomplex characteristics that improve intracellular delivery, rather than differences in the inherent siRNA activity of siLDHC#1 and siLDHC#2. Furthermore, the knockdown efficiency of the CPP:siLDHC complexes was confirmed at the protein level with the 10R-RGD:siLDHC#2 and cRGD-10R:siLDHC#2 complexes showing the highest efficiencies, reducing LDHC protein expression by approximately 40% (Figure 5C).

**Figure 6:**
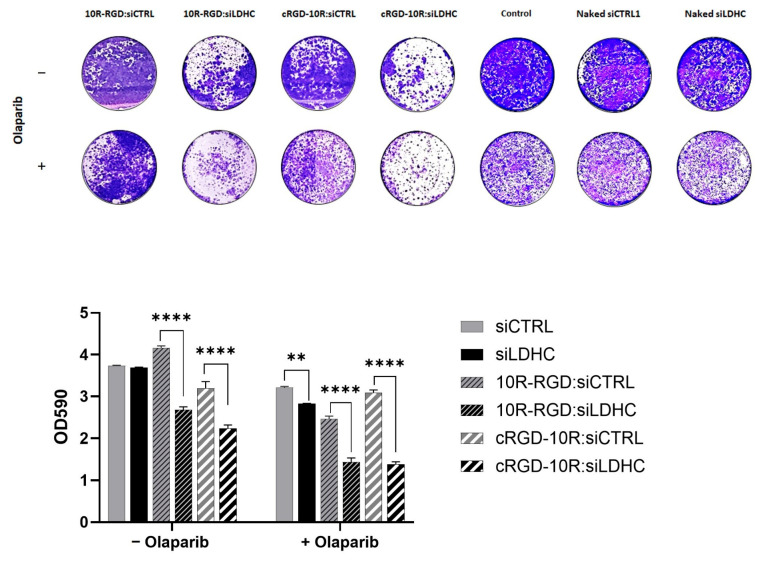
CPP:siLDHC treatment significantly reduces the clonogenic ability of MDA-MB-468 cells and potentiates olaparib treatment. Representative images of clonogenic assay using 10R-RGD:siRNA and cRGD-10R:siRNA alone or in combination with olaparib short-term treatment (72 h). Bar chart depicting crystal violet absorbance measurements from three independent experiments (mean ± SEM). Statistical analysis was performed using the one-way ANOVA test with Šídák correction. ** *p* < 0.01, **** *p* < 0.0001.

The clonogenic assay was used to evaluate the effects of CPP-mediated *LDHC* silencing, alone or in combination with olaparib, on the long-term survival of MDA-MB-468 cells. Based on the LDHC mRNA and protein knockdown results, the 10R-RGD:siRNA and cRGD-10R:siRNA complexes with the highest silencing efficiency were selected for further functional validation. In accordance with our previous observations [10], CPP-mediated silencing of *LDHC* alone significantly reduced the clonogenic ability of MDA-MB-468 triple-negative breast cancer cells, and improved the treatment response to olaparib (Figure 6). This decrease in clonogenicity highlights the therapeutic potential of targeting LDHC in TNBC.

### 3.4. 10R-RGD and cRGD-10R:siRNA Complexes Exhibit Anti-Tumor Activity with Minor Toxicity in Breast Cancer Zebrafish Xenograft Model

**Figure 7:**
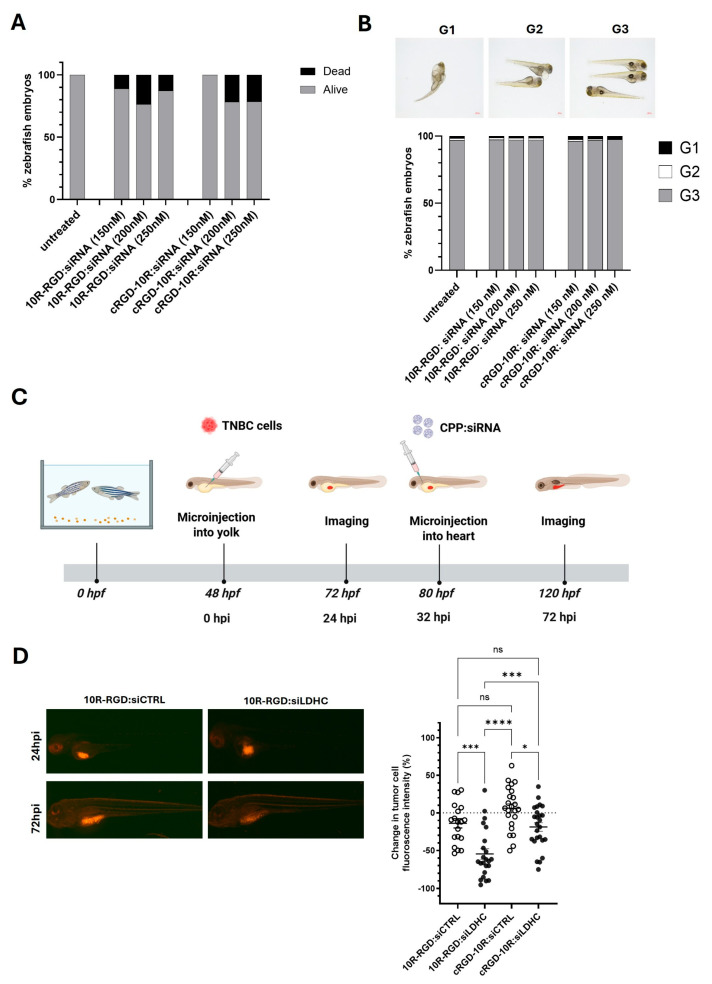
10R-RGD and cRGD-10R:siRNA complexes exhibit anti-tumor effects in TNBC zebrafish xenograft model without inducing toxicity. (**A**) Zebrafish mortality and (**B**) morphological abnormalities following treatment with 10R-RGD:siRNA and cRGD-10R:siRNA complexes. G1: severely affected, G2: mildly affected, G3: normal. (**C**) Diagram of TNBC zebrafish xenograft model, depicting TNBC cell injection in the yolk at 2 days post-fertilization (dpf) and CPP:siRNA intracardial treatment at 32 h post-injection (hpi). (**D**) Representative images of fluorescent tumor cells at 24 hpi and 72 hpi. Change in tumor cell burden, measured as % change in fluorescence intensity following CPP:siRNA treatment. Scatter dot plots represent fluorescence intensity values from two independent experiments (mean ± SEM). Statistical analysis was performed using a one-way ANOVA test with Tukey’s multiple testing correction. * *p* < 0.05, *** *p* < 0.001, **** *p* < 0.0001.

Our promising in vitro results, showing good LDHC knockdown efficiency, reduced colony-forming ability, and low cytotoxicity, prompted us to evaluate the toxicity of the CPP:siRNA nanocomplexes in vivo. Cytotoxicity was assessed through microinjection of 10R-RGD:siRNA and cRGD-10R:siRNA complexes (150, 200, and 250 nM) into single-cell stage wild-type AB zebrafish embryos. Minor toxicity was observed for both CPP:siRNA complexes at various doses (Figure 7A). No significant morphological abnormalities of the zebrafish embryos were observed after microinjection with either 10R-RGD:siRNA or cRGD-10R:siRNA complexes (Figure 7B).

Next, the therapeutic potential of CPP-based siLDHC delivery was assessed using a TNBC zebrafish xenograft model (Figure 7C). We selected the MDA-MB-468 cell line as a proof-of-concept TNBC model based on its positive integrin αVβ3 expression and higher expression of LDHC compared to the BT-549 cell line [14]. Treatment of MDA-MB-468 zebrafish xenograft larvae with 10R-RGD and cRGD-10R:siLDHC nanocomplexes resulted in a significant reduction in tumor burden (Figure 7D), achieving up to 50% reduction in tumor size using the 10R-RGD complex. These findings are in line with our in vitro results, which demonstrate higher cellular uptake and *LDHC* knockdown efficiency using 10R-RGD:siRNA complexes compared to cRGD-10R:siRNA. Of note, the 10R-RGD:siLDHC more greatly reduced tumor burden as compared to cRGD-10R:siLDHC, while no significant differences were found between the 10R-RGD:siCTRL and cRGD-10R:siCTRL.

## 4. Discussion

Over the last few decades, cancer treatment has made great strides with targeted therapy and immunotherapy improving the prognosis of patients with various cancer types. Targeted therapy with mTOR, CDK4/6, or PI3K inhibitors in combination with hormonal therapy has now entered the clinic for the treatment of advanced breast cancer patients with hormone receptor-positive tumors [23]. Further, patients with Her2-enriched breast tumors benefit from combination treatment of chemotherapy with a variety of anti-Her2 treatment modalities [24]. Despite these advances in molecular targeting, treatment options for patients with triple-negative breast cancer remain limited. While TNBC patients with *BRCA1*/*2* mutations may benefit from PARP inhibitors, and the prognosis of patients with PD-L1 tumor expression may be improved by immune checkpoint blockade, the majority of TNBC patients still receive standard-of-care chemotherapy alongside radiotherapy and surgery [2,25]. Moreover, while TNBC patients often exhibit good pathological response rates following chemotherapy, their overall prognosis remains poor due to early cancer recurrence and the development of chemoresistance. Hence, there is an unmet need to find novel therapeutic targets to increase their clinical outcome.

Previously, we found that targeting LDHC, a highly tumor-specific metabolic enzyme, significantly reduces genomic integrity, impairs the clonogenic ability of breast tumor cells, and enhances treatment responses to DNA damage repair-related drugs in vitro. Hence, we hypothesized that targeting of LDHC could be used to complement traditional cancer treatments to enhance anti-tumor efficacy. However, specific LDHC inhibitors and blocking antibodies are currently unavailable. Therefore, we explored the therapeutic potential of siRNA-based drugs to target LDHC tumor expression. To date, five siRNA-based drugs have been approved by the FDA for the treatment of liver diseases, of whom four utilize N-acetylgalactosamine (GalNAc) as a targeting ligand for the asialoglycoprotein receptor (ASGPR), predominantly expressed in hepatocytes [26]. Although no siRNA therapeutics have been approved for cancer treatment to date, numerous candidates are currently in phase I/II clinical trials [26].

In the present study, we used cell-penetrating peptides as a delivery system for LDHC siRNA in triple-negative breast cancer cells. More specifically, we explored the use of the integrin αVβ3 RGD binding motif to facilitate tumor homing and penetrance of the siRNA therapeutic. Four CPPs; R10, 10R-RGD, cRGD-10R, and iRGD-10R; were studied for siRNA complexing efficiency, serum stability, and cytotoxicity in vitro. All four CPPs formed uniform structures with the siRNA molecules, resulting in positively charged nanocomplexes that enhance permeability and retention [27]. Complexing siRNA with the CPPs enhanced their persistence in human serum, indicating that the peptides were able to protect the siRNA from circulating RNA enzymes. Furthermore, only minor toxicity was observed using the CPP:siRNA complexes in either breast cancer cells (αVβ3 negative or positive TNBCs) or non-cancerous cells (integrin αVβ3 positive IMR-90 and HUVEC cells), indicating a favorable safety profile with no adverse side effects. Comparative analysis of LDHC knockdown efficiency revealed that the 10R-RGD:siLDHC and cRGD-10R:siLDHC complexes more effectively reduced LDHC expression in MDA-MB-468 triple-negative breast cancer cells (integrin αVβ3 positive), achieving up to a 40% reduction at the protein level and approximately 60% at the mRNA level. These findings are in accordance with other preclinical studies reporting comparable silencing efficiencies using RGD-based CPPs in cancer. For instance, cRGD-mediated siRNA delivery reduced protein Lamin A/C expression by 40% in the triple-negative breast cancer cell line MDA-MB-231 [28]. Targeting of PIK3CB by a bivalent cRGD-siRNA complex decreased protein expression by 30–40% in the U87MG glioblastoma cell line, and reduced tumor growth in an orthotopic glioblastoma xenograft model [29].

Next, we evaluated the therapeutic potential of targeting LDHC in TNBC using CPP-siRNA-based therapy. Based on our previous work, we investigated whether CPP:siLDHC treatment alone and in combination with olaparib impacts the clonogenic ability of TNBC cells. Similarly to our previous observations, CPP:siRNA-based targeting of LDHC, in particular using 10R-RGD:siLDHC and cRGD-10R:siLDHC, significantly reduced tumor cell clonogenic ability and greatly enhanced the anti-tumor effect of olaparib, indicating that CPP:siLDHC therapy could provide a promising strategy for the treatment of TNBC. To validate the clinical significance of CPP:siLDHC-based therapy, the anti-tumor activity and safety profiles of 10R-RGD:siLDHC and cRGD-10R:siLDHC delivery were investigated in a TNBC zebrafish xenograft model. Both complexes significantly reduced tumor burden, with the 10R-RGD complex achieving up to a 50% reduction in tumor size, without inducing major morphological abnormalities or death. In comparison, iRGD-polymersomes targeting VEGF in breast cancer xenograft mice inhibited tumor growth by 67% [30], and cRGD-PEG-MAL:siRNA targeting VEGFR2 in NSCLC xenograft mice reduced tumor volume by approximately 70–90% [31], demonstrating that our CPP:siLDHC system achieves robust in vivo efficacy in zebrafish relative to reported mammalian models. While these findings indicate that LDHC targeting using CPPs significantly reduces tumor burden in zebrafish xenografts, future studies are needed to validate their efficacy and safety in a mammalian organism. This is of particular importance to evaluate the therapeutic effect of the complexes in the presence of a fully functional adaptive immune response, given that we previously demonstrated that *LDHC* silencing improves anti-tumor immunity through modulation of cytokine production and dysregulation of immune checkpoint signaling [32]. In addition, a comprehensive toxicological analysis of major organs in a mammalian model will provide further insights into the safety of our CPP-based approach. Furthermore, future modifications of our CPP:siLDHC nanocomplexes using advanced nanocarriers (extracellular vesicles, inorganic nanoparticles, or polymerosomes), stimuli-responsive elements (ROS- or pH-sensitive moieties), or stability-enhancing features (PEG, chitosan, or histidine domains) may further improve therapeutic efficacy in vivo [33,34,35,36,37,38,39,40,41,42,43,44,45,46].

## 5. Conclusions

In conclusion, we identified two CPP:siRNA nanocomplexes, 10R-RGD:siLDHC and cRGD-10R:siLDHC, that persist in serum and effectively target LDHC in triple-negative breast cancer, exhibiting significant anti-tumor activity and a favorable safety profile in vitro and in vivo. These findings corroborate LDHC as a promising target for TNBC therapy and highlight the potential of CPP:siRNA-based drugs as a novel therapeutic approach for cancer therapy.

## 6. Patents

Julie Decock and Adviti Naik are named inventors on patent application WO2021251842A1, which is assigned to Qatar Foundation. The authors declare that this did not influence the design, execution, interpretation, or reporting of the study.

## Figures and Tables

**Table 1:** The biochemical properties of CPPs.

CPP	Sequence	Length	Molecular Weight (MW)	Purity %
R10	RRRRRRRRRR	10	1579.9	98
10R-RGD	RRRRRRRRRR-**RGD**	13	1908.23	96
cRGD-10R	DGARYC**RGD**CFDG-RRRRRRRRRR	23	2994.39	99
iRGD-10R	C**RGD**KGPDCRRRRRRRRRR	19	2509.93	99

## Data Availability

The original contributions presented in this study are included in the article/supplementary material. Further inquiries can be directed to the corresponding author.

## Supplementary Materials

The following supporting information can be downloaded at: https://www.mdpi.com/article/10.3390/pharmaceutics18010078/s1, Figure S1: Characterization of integrin αVβ3 expression across four breast cancer and two non-cancerous cell lines.

## Abbreviations

CPP	cell-penetrating peptide
CTA	cancer testis antigen
Her2	Human Epidermal Growth Factor Receptor 2
LDHC	Lactate Dehydrogenase C
TNBC	triple-negative breast cancer
