# Supplementary figures and images for: Cell-Penetrating Peptide-Mediated siRNA Targeting of LDHC Suppresses Tumor Growth in a Triple-Negative Breast Cancer Zebrafish Xenograft Model
Source: Pharmaceutics. 2026 Jan 7;18(1):78. doi: 10.3390/pharmaceutics18010078 (PMC12845483; doi:10.3390/pharmaceutics18010078)

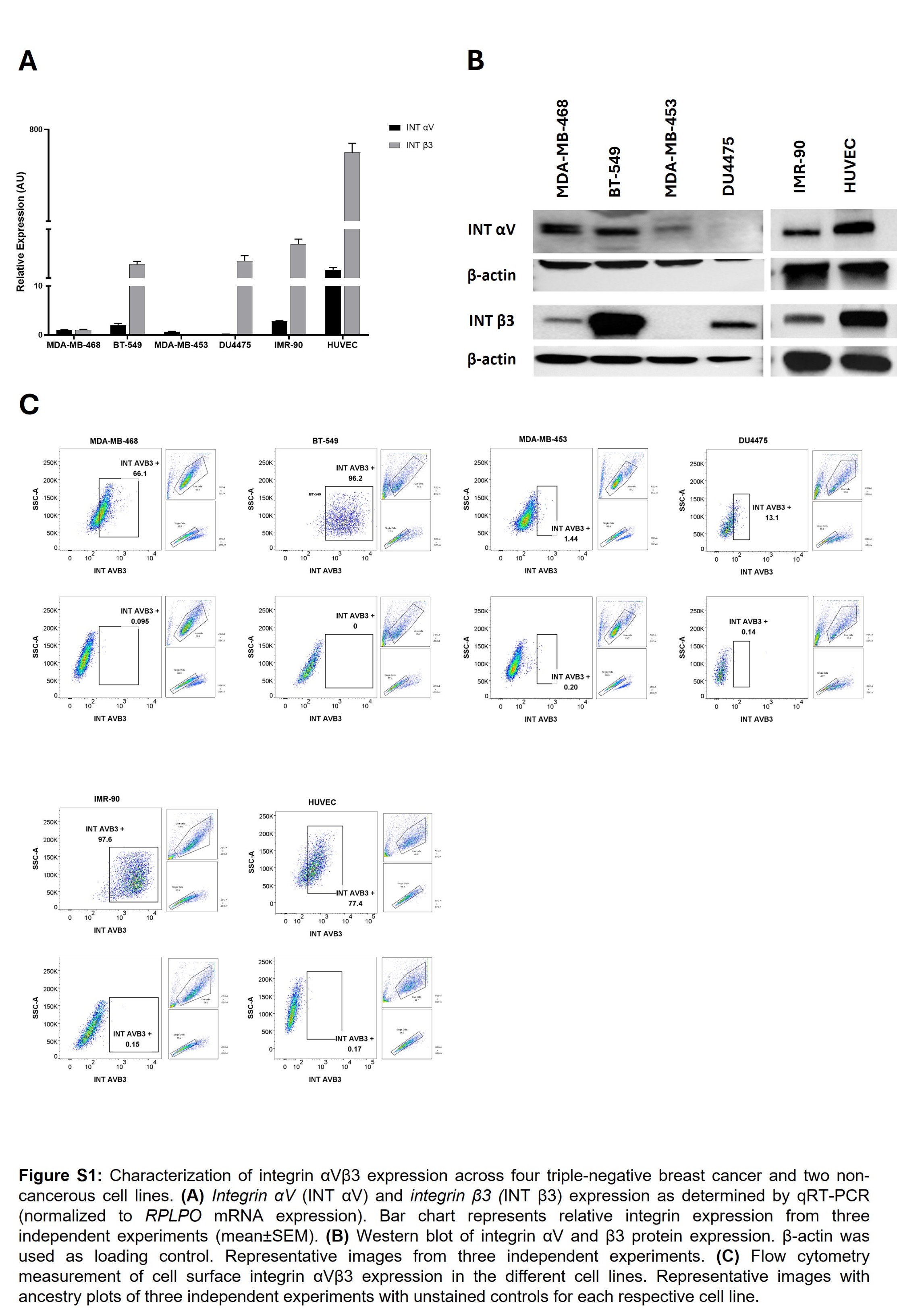

Supplement: Supplementary file 1 [file pharmaceutics-18-00078-s001.zip › pharmaceutics-4057178-supplementary.jpg]
